# Multi-sensor ecological momentary assessment of behavioral and psychosocial predictors of weight loss following bariatric surgery: study protocol for a multicenter prospective longitudinal evaluation

**DOI:** 10.1186/s40608-018-0204-6

**Published:** 2018-11-05

**Authors:** Stephanie P. Goldstein, J. Graham Thomas, Sivamainthan Vithiananthan, George A. Blackburn, Daniel B. Jones, Jennifer Webster, Richard Jones, E.Whitney Evans, Jody Dushay, Jon Moon, Dale S. Bond

**Affiliations:** 1 Weight Control and Diabetes Research Center, Department of Psychiatry and Human Behavior, The Miriam Hospital/Warren Alpert Medical School of Brown University, 196 Richmond Street, Providence, RI 02909 USA; 2 Department of Surgery, The Miriam Hospital/Warren Alpert Medical School of Brown University, 195 Collyer Street, Providence, RI 02904 USA; 3 Beth Israel Deaconess Medical Center, Department of Surgery, Center for the Study of Nutrition Medicine, Feldberg 880, East Campus, 330 Brookline Avenue, Boston, MA 02215 USA; 4 Department of Psychiatry and Human Behavior, Warren Alpert Medical School of Brown University, Butler Hospital, 345 Blackstone Boulevard, Box G-BH, Providence, RI 02906 USA; 5 Department of Medicine, Division of Endocrinology, Beth Israel Deaconess Medical Center, Feldberg 880, East Campus, 330 Brookline Avenue, Boston, MA 02215 USA; 6 MEI Research, Ltd, 6016 Schaefer Road, Edina, MN 55436 USA

**Keywords:** Bariatric surgery, Weight loss, Obesity, Ecological momentary assessment, Diet, Physical activity, Technology

## Abstract

**Background:**

Bariatric surgery is currently the most effective strategy for producing significant and durable weight loss. Yet, not all patients achieve initial weight loss success and some degree of weight regain is very common, sometimes as early as 1–2 years post-surgery. Suboptimal weight loss not fully explained by surgical, demographic, and medical factors has led to greater emphasis on patient behaviors evidenced by clinical guidelines for appropriate eating and physical activity. However, research to inform such guidelines has often relied on imprecise measures or not been specific to bariatric surgery. There is also little understanding of what psychosocial factors and environmental contexts impact outcomes. To address research gaps and measurement limitations, we designed a protocol that innovatively integrates multiple measurement tools to determine which behaviors, environmental contexts, and psychosocial factors are related to outcomes and explore how psychosocial factors/environmental contexts influence weight. This paper provides a detailed description of our study protocol with a focus on developing and deploying a multi-sensor assessment tool to meet our study aims.

**Methods:**

This NIH-funded prospective cohort study evaluates behavioral, psychosocial, and environmental predictors of weight loss after bariatric surgery using a multi-sensor platform that integrates objective sensors and self-report information collected via smartphone in real-time in patients’ natural environment. A target sample of 100 adult, bariatric surgery patients (ages 21–70) use this multi-sensor platform at preoperative baseline, as well as 3, 6, and 12 months postoperatively, to assess recommended behaviors (e.g., meal frequency, physical activity), psychosocial indicators with prior evidence of an association with surgical outcomes (e.g., mood/depression), and key environmental factors (e.g., type/quality of food environment). Weight also is measured at each assessment point.

**Discussion:**

This project has the potential to build a more sophisticated and valid understanding of behavioral and psychosocial factors contributing to success and risk after bariatric surgery. This new understanding could directly contribute to improved (i.e., specific, consistent, and validated) guidelines for recommended pre- and postoperative behaviors, which could lead to improved surgical outcomes. These data will also inform behavioral, psychosocial, and environmental targets for adjunctive interventions to improve surgical outcomes.

**Trial registration:**

Registered trial NCT02777177 on 5/19/2016.

**Electronic supplementary material:**

The online version of this article (10.1186/s40608-018-0204-6) contains supplementary material, which is available to authorized users.

## Background

Over the past 15 years, bariatric surgery has amassed a strong evidence base as a first-line treatment for severe obesity [1, 2]. Approximately 468,609 surgeries are performed worldwide each year [3]. The most common procedures are sleeve gastrectomy (SG) and Roux-en-Y gastric bypass (RYGB). Bariatric surgery involves anatomical changes, as well as neural and hormonal shifts that facilitate weight loss through changes in energy balance, metabolism, satiety and appetite, and disease processes [4]. As such, surgical procedures can also lead to remission or resolution of obesity co-morbidities (e.g., Type 2 diabetes) and restore health-related quality of life [5]. Bariatric surgery produces approximately 30% weight loss over a period of 6–7 years [6, 7]. However, there is substantial individual variability in short- and long-term weight loss [6, 7].

While several factors have been shown to influence weight loss outcomes (e.g., procedure, baseline weight, age, and race), energy balance behaviors and related psychosocial factors are of considerable interest given their amenability to change and potential to enhance surgical effects [8]. As such, various clinical guidelines have been put forth to describe recommended eating and physical activity behaviors to maximize surgical benefits [9]. Examples of dietary recommendations include ≥ 5 recommended meals/snacks per day of ≤ 8 oz, ≥ 5 servings of fruits and vegetables daily, and ≥ 20-min duration of meals/snacks. Guidelines also suggest ≥ 30 min of daily physical activity.

At present, development of evidence-based behavioral guidelines for the bariatric surgery population is challenging due to relative lack of prospective observational and experimental research and limitations associated with traditional measurement methodologies. Unfortunately, there is little research focused on bariatric surgery patients specifically, making the formation of consistent, empirically-supported behavioral guidelines for this population difficult. Compounding these concerns, there is a paucity of research examining compliance with recommended eating and physical activity behaviors. Moreover, the few published studies have relied primarily on retrospective chart reviews, self-report questionnaires, and clinical interviews. These traditional methodologies are known for biases that reduce validity and reliability of information collected. For example, research comparing self-reported to objectively-measured physical activity and sedentary behavior revealed that bariatric surgery patients typically self-report postoperative increases in physical activity that are not supported by objective measurements [10, 11]. Additionally, traditional methodologies do not collect data with the level of detail, number of repeated observations, or environmental context that would allow for precise estimates of behavior and associated variability.

To this end, innovative measurement strategies that maximize data quality are needed to study behavioral, psychosocial, and environmental contributors to postoperative weight loss, thus providing a more rigorous evidence base for pre- and postoperative clinical guidelines and interventions [12]. One potential solution is ecological momentary assessment (EMA), a method by which participants are prompted to give in-the-moment reports on selected behaviors, cognitive/emotional states, and environmental conditions several times throughout the day (usually using mobile phones) [13]. Not only does EMA capitalize on external validity by assessing constructs in the environment as they occur naturally, but it also eliminates the need for retrospective self-report, thereby removing many sources of bias [14, 15]. The power of EMA data can be strengthened further using a multi-method measurement approach [12]. The recent rise of unobtrusive, wearable sensors that connect to smartphones in real-time makes it possible to obtain continuous, objective behavioral measurements in an individual’s natural environment.

We have successfully employed both EMA and objective sensors (separately, but not in combination) to investigate adherence to postoperative guidelines. EMA of physical activity intentions and behavior demonstrated that participants are rarely fulfilling their intentions to exercise and these intentions are not consistent with established guidelines [16]. Our use of EMA to measure eating behavior revealed that participants refrained from drinking while eating and took vitamin supplements and medication as prescribed, but they were not generally adherent with the remainder of postoperative guidelines for eating [17]. These studies indicate that EMA and objective sensors can facilitate a deeper understanding of eating and physical activity behaviors, and the contexts in which they occur, that can better inform postoperative guidelines as well as behavioral interventions to improve adherence to such guidelines [18].

While our preliminary studies indicated that mobile health (mHealth) technology (i.e., use of smartphones for self-report surveys, wearable sensors) is a promising avenue to measure eating and physical activity behaviors of bariatric surgery patients, there remains several key areas for growth. First, the use of an *integrated* mHealth system is warranted, as research to date has only employed these methods separately within the bariatric population. By combining EMA with objective sensors, it is possible to obtain more valid and reliable estimates of behavioral patterns that can be further enriched by additional contextual information. For example, accelerometry, a well-accepted method for objectively assessing physical activity, can be enhanced with EMA questions delivered by smartphone to assess type of exercise, context, motivational factors, and barriers (all of which accelerometry alone cannot provide). Second, little attention has been devoted to examining whether compliance with published behavioral recommendations relates to postoperative weight loss outcomes overall, and specifically the intervals during which compliance may have the greatest impact. Third, there has been little consideration given to whether important psychosocial aspects (e.g., mood, disinhibition, cognitive restraint) and/or environmental factors (e.g., location of eating, availability of foods) may predict weight loss outcomes via influence on pre- and/or postoperative behavioral compliance.

Recognizing the aforementioned gaps in research on behavioral and psychosocial predictors of bariatric surgery outcomes, the NIH called for projects to address the problem and funded the project described herein to address the following aims: (1) assess the feasibility and acceptability of using a multi-sensor mHealth platform to collect data in real-time on behavioral and psychosocial predictors of weight loss outcomes; (2) evaluate which behavioral and psychosocial factors predict outcomes and the times at which each factor has the strongest effect; and (3) identify causal pathways by which psychosocial factors influence outcomes via effects on behavior, as well as moderators that explain for whom and under what conditions the influence is the strongest. The following sections describe the overall study design, with an emphasis on the multi-sensor mHealth platform, given its novelty and innovation. Additionally, challenges and considerations in developing and deploying the platform that are representative of those encountered in mHealth studies more broadly, are discussed.

## Methods

The project involves a prospective cohort study designed to evaluate predictors of weight loss after bariatric surgery, including energy balance behaviors (i.e. physical activity, sedentary behavior, and eating behavior), psychosocial factors (e.g., appetite/motivation to eat, physical and social cues), and environmental factors (e.g., availability of food). In particular, we aim to improve our understanding of the associations between weight loss and behaviors targeted by postoperative guidelines so that the guidelines can be made more specific and consistent. Participants (target *n* = 100) with severe obesity (body mass index ≥35 kg/m^2^) are recruited prior to undergoing bariatric surgery with predictor variables measured 3 to 8 weeks preoperatively (baseline) and at 3, 6, and 12 months postoperatively. As described in greater detail below, we configured an established EMA platform to allow for integration of: 1) direct, sensor-based measures of energy balance behaviors (i.e. ActiGraph Link (ActiGraph, LLC, Pensacola, FL, USA)) to measure physical activity, sedentary behavior and sleep; Bite Counter ((Bite Technologies, Pendelton SC, USA) to measure eating behavior), and 2) self-report surveys administered several times daily via smartphone to capture subjective reports of other behaviors and experiences. These data are further supplemented with phone-based 24-h dietary recalls, paper-and-pencil questionnaires, and chart reviews. Weight is measured at all assessment points.

### Setting

This study is taking place at two university-based hospital bariatric surgery centers, The Miriam Hospital (Providence, Rhode Island, USA) and the Beth Israel Deaconess Medical Center (Boston, Massachusetts, USA).

### Participants

A target sample of 100 participants is enrolled on a rolling basis. Eligibility is limited to individuals greater than 21 years of age with severe obesity (body mass index ≥35 kg/m^2^) who are undergoing RYGB or SG at either study site. Individuals are excluded from participating if they: 1) are currently involved in a weight loss or related behavioral form of treatment outside the context of standard surgical care (patient support groups, education, and pre- and postoperative dietary counseling are considered standard surgical care); or 2) report a condition that in the opinion of the investigators would preclude adherence to the measurement protocol, primarily including plans to relocate geographically, substance abuse or other significant uncontrolled psychiatric problem, or terminal illness. The above inclusion/exclusion criteria are designed to identify a heterogeneous sample of patients, ensure maximum generalizability to the national bariatric surgery population, and provide data on behavioral and psychosocial outcomes of interest.

### Procedure

This study was approved by The Miriam Hospital Institutional Review Board (Version 2.0 July 2017). Protocol modifications were submitted to this IRB for approval. The Miriam Hospital IRB requires that modifications include plans for notifying participants should the modification impact their study participation. There were no modifications of the current protocol requiring notice to participants, funding agency, or trial registries. An additional file details the informed consent protocol approved by The Miriam Hospital IRB (see Additional file 1).

**Fig. 1:**
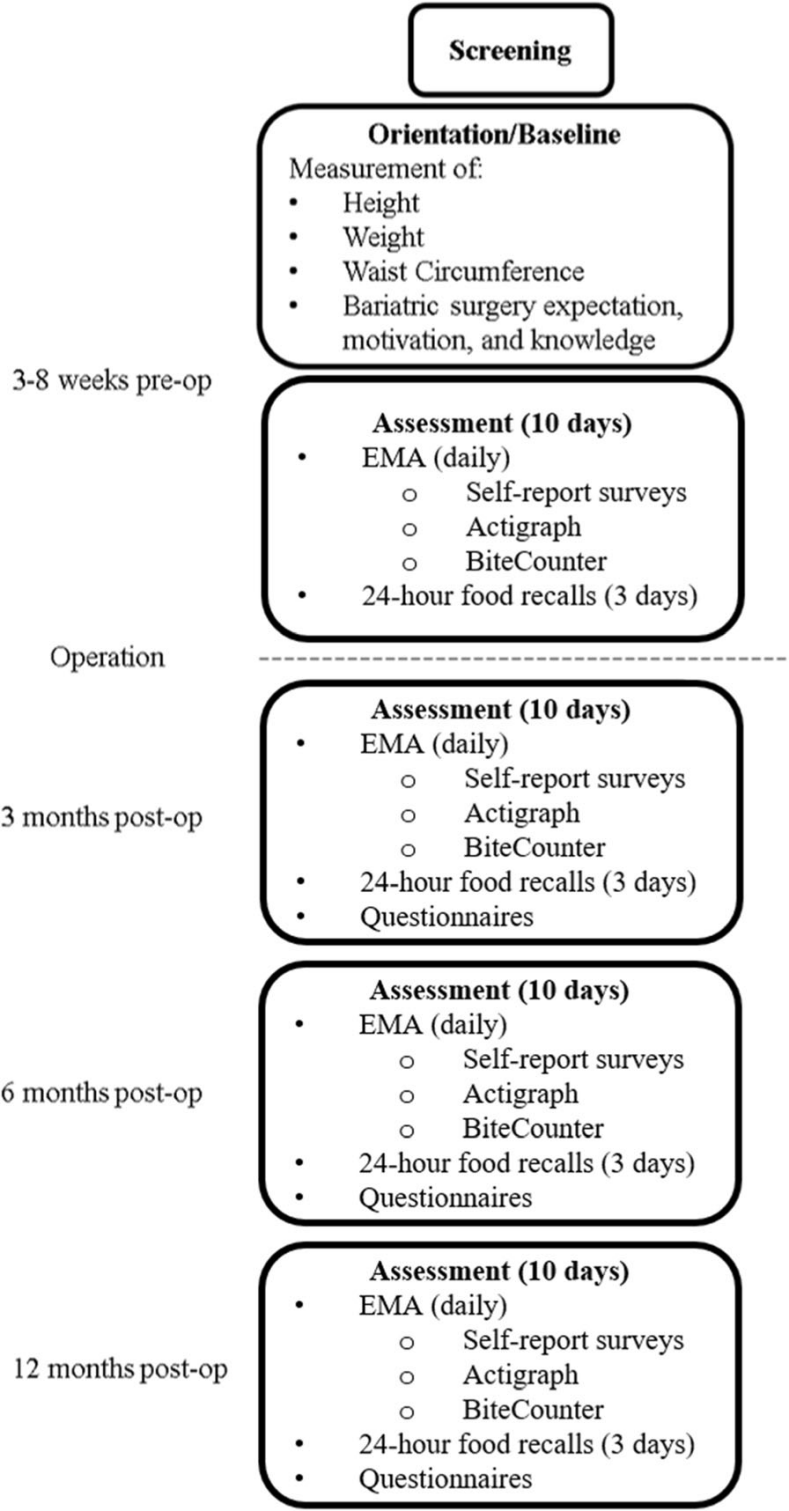
Study Timeline

See Fig. 1. Study Timeline for a study schematic. Patients from both sites are recruited 3 to 8 weeks preoperatively during a regularly scheduled clinic visit. At these visits, a surgeon or another provider and member of the surgical team provides patients with a flyer describing the study. Patients who wish to be contacted further about the study provide a signature and contact number to the staff. A bariatric surgery team member faxes this information to the appropriate research center so that research staff can conduct a brief screening by phone and schedule an in-person orientation/baseline study visit.

At the in-person initial orientation/baseline visit participants provide informed consent with a trained member of the study staff, have height, weight and waist circumference measured by trained research staff, complete questionnaires, are shown how the 24-h dietary recalls will be completed, and receive the EMA equipment described below (Android smartphone, ActiGraph Link, and Bite Counter). Participants then receive training in how to wear the sensor devices and complete self-report surveys using electronic forms on the smartphone.

Upon completion of training, participants begin their first 10-day EMA assessment period. A 10-day period is consistent with prior EMA studies and balances participant burden with the need to measure each key construct multiple times at each assessment period over weekdays and weekends [13, 19]. At all subsequent postoperative assessments (i.e., 3, 6, and 12 months), participants return to the research centers to complete questionnaires, receive a refresher on the EMA protocol, and then complete the protocol for 10 days. Anthropometric measurements (i.e., height, weight, body mass index, waist circumference, weight loss) are obtained by trained research staff at all the above time points. Participants receive $75 at the end of each assessment and can earn 50 cents for each survey completed via smartphone. This extra compensation of 50 cents per survey adds up to about $25 during each 10-day assessment if the participant completes about 80% of the surveys. Participants with good compliance are therefore expected to earn a total compensation of about $100 for each 10-day assessment. The smartphone used for EMA automatically tracks and displays participants’ compliance with prompted self-report surveys, which is a novel and innovative method to encourage high compliance in a research setting. Real-time EMA data are supplemented with assessment of dietary intake, paper-and-pencil questionnaires, chart review, and anthropometric measures to establish a comprehensive record of pre- and postoperative patterns. Below, we first describe the PiLR Health System and procedure for delivering smartphone surveys. Then, we describe the study measures (i.e., the objective sensors and constructs assessed with smartphone self-report surveys).

### PiLR EMA system

PiLR Health™ is a platform for mobile assessment and intervention that has been developed and is maintained by MEI Research, Ltd. through grants and contracts from multiple NIH institutes. We collaborated with MEI to customize the platform to execute the study procedures. The PiLR platform used for the current study consists of a smartphone-based application, or “hub”, that operates on Android devices and cloud servers. Using its always-on Internet connection, the smartphone hub receives instructions from, and transmits its data to, a study server that coordinates EMA implementation and data integration for the study. The server is accessible via a Web-based interface that allows the research team to implement the EMA protocol (e.g., load surveys, define participant engagement periods, assign devices to participants, assign sensors), view summary reports (e.g., real-time participant compliance with the EMA protocol), and retrieve data files. The smartphone native app is configured for store-and-forward communications so that it functions independently if Internet connectivity is interrupted.

**Fig. 2:**
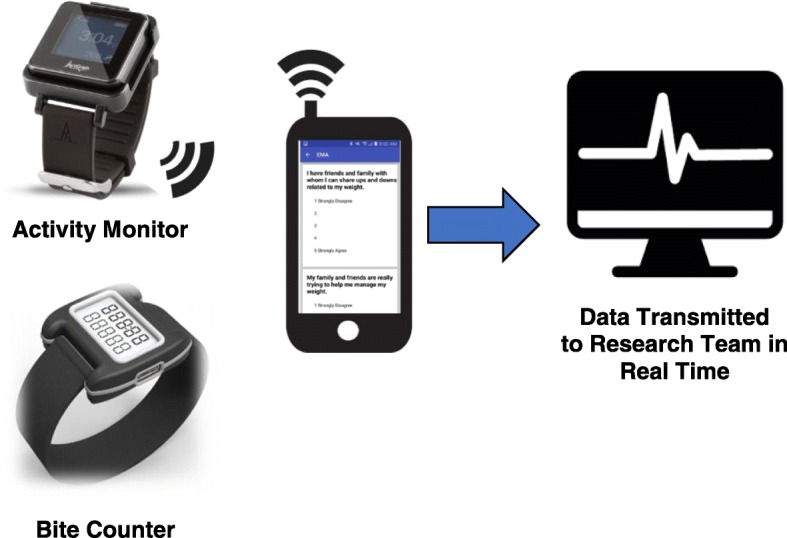
EMA System and Components

See Fig. 2. EMA System and Components for a depiction of the multi-sensor measurement tools. The EMA components used in the current study are a wrist-worn accelerometer (ActiGraph Link; ActiGraph, LLC, Pensacola, FL, USA) to detect physical activity, sedentary behavior, and sleep; a wrist-worn device to monitor eating (Bite Counter; Bite Technologies, Pendelton SC, USA), and smartphone-based self-report surveys. These measurement tools were chosen based on their methodological rigor and feasibility for use in the bariatric surgery population.

The PiLR platform was chosen for this study for specific advantages related to maximizing data quality and assessment that allow us to achieve the study aims. First, we can check the quality of the data during collection to ensure compliance with the EMA protocol and detect any errors that could occur. In this project, research staff review the first 2 days of participants’ smartphone self-report surveys and sensor data remotely to check adherence and confirm that data are being collected and received as expected. If adherence to prompted self-report surveys is less than 90% or there are problems with data collection from any of the devices (e.g., the participant is not wearing devices, or the data are not being received), the participant is contacted by phone to resolve the problem. Those first 2 days of EMA can then be excluded from analysis to account for reactivity (i.e., change in behavior occurring at EMA initiation, before it becomes routine). Second, the system automatically monitors data quality in real-time throughout the remainder of the study period. For example, PiLR Health alerts the research team and participant if cumulative adherence to prompted self-report surveys falls below 85%, or if the sensor devices are not worn during waking hours for ≥4 h. The research team can then contact the participant by phone to resolve the problem(s) if they persist beyond the end of the day.

To limit participant burden and improve data quality, EMA self-report surveys can be programmed so that they are adaptive by selecting questions based on prior responses earlier in the survey (e.g., if participants indicate early in a survey that eating has not occurred recently, they are asked no more questions about eating in that survey and are instead asked about other, more relevant behaviors or experiences). The PiLR platform used in this study extends that concept by capitalizing on sensors data to determine when certain surveys are triggered. We use the objective sensors in the current project not only as assessment tools, but also to prompt completion of “in-the-moment” self-report surveys about experiences related to physical activity and sedentary behavior that cannot be measured via sensor (e.g., type of physical activity, location of activity).

### Smartphone self-report surveys

All participants are provided with an Android device (Samsung Galaxy S7; Samsung Electronics, South Korea) running the mobile application described above. Participants are asked to respond to “in-the-moment” self-report surveys on this device for 10-day assessment periods. Participants are prompted via vibration, an audible tone, and a message on the smartphone screen to complete real-time self-report surveys several times per day. A beginning-of-day survey is initiated at 8:00 a.m. Prompts to rate eating, physical activity, and sedentary behavior are initiated when specific events are detected via objective monitoring, as described above. Key psychosocial and environmental variables are rated during these prompts and at 4 semi-random prompts anchored at 11:00 a.m., 2:00 p.m., 5:00 p.m., and 8:00 p.m. Participants are asked to self-initiate an end-of-day survey “before bed”. All prompted surveys are capped at 12 per day to limit participant burden [13, 19]. Below we describe the content of self-report surveys.

## Measures

See Table 1 for a summary of all behavioral, psychosocial and environmental predictors assessed in this study with related assessment methods.

**Table 1:** Behavioral, Psychosocial, and Environmental Predictors and Related Assessment Methods

Predictor	Assessment Method
Physical activity	
Level (light-vigorous), duration	ActiGraph Link
Total & active energy expenditure (kcal/d)	ActiGraph Link
Steps and total distance/day	ActiGraph Link
Types of PA	EMA
PA barriers and intentions	EMA
Sedentary behavior	
Total minutes/day and % time	ActiGraph Link
Types of sedentary behavior	EMA
Eating behavior	
Frequency, timing, duration, rate, and volume	Bite Counter
Total energy intake, diet composition, & quality	Dietary Recall
Hunger and satiety	EMA
Appetite/motivation to eat	EMA
Binge eating & loss of control	EMA
Planned eating	EMA
Grazing	EMA
Dietary restraint and disinhibition	EMA
Behavioral complications	EMA
Sleep habits (total time and efficiency)	ActiGraph Link
Other adherence behaviors	
Self-weighing	EMA
Attendance at clinical follow-ups	Chart Review
Adherence to medications/vitamins	EMA
Psychosocial factors	
Mood, stress, energy, fatigue	EMA
Health locus of control	EMA
Social support	EMA
Outcomes expectations	Questionnaires
Bariatric surgery motivation & satisfaction	Questionnaires
Understanding of behavioral recommendations	Questionnaires
Environmental factors	
Exposure to and availability of palatable foods	EMA
Cues for eating, activity, and sedentariness	EMA
Eating location/setting & behavior/proximity of others	EMA

### Physical activity, sedentary behavior, and sleep

The ActiGraph GT9X Link (ActiGraph, LLC, Pensacola, FL, USA) objectively assesses daily time spent in physical activity, sedentary behaviors, and sleep. This device employs rigorously validated triaxial accelerometer and proprietary data filtering technology used in previous generation devices to reliably estimate free-living activity in adult populations, including those undergoing bariatric surgery [20, 21]. The ActiGraph Link is equipped with a sensor on the back of the device that automatically detects when the device has been removed to assist with compliance monitoring. The ActiGraph Link is also thought to be more tolerable to participants, as compared to waist-worn monitors or armbands, because it is specifically designed to appear and feel like a wrist watch.

Participants are instructed to wear the ActiGraph Link on their non-dominant wrist 24 h per day, exclusive of bathing and swimming, for the 10-day EMA protocol at the 4 assessment periods. For each of the 10-day assessments, a participant’s data is considered valid if he or she wears the device for ≥10 h on ≥ 5 days (including ≥1 weekend day). The number of minutes per day participants spend in sedentary behavior and physical activity of different intensities is being determined using metabolic equivalents (METs), with activities < 1.5 METs, activities1.5–2.9 METs, activities 3.0–5.9 METs, and activities ≥ 6.0 METs classified as sedentary, light, moderate, and vigorous, respectively. We are particularly interested in moderate-to-vigorous physical activity (MVPA) and bout-related MVPA (≥ 10 min of activity at a time) given that this level of physical activity is emphasized in recommendations for both bariatric surgery patients and the general adult population [22–24]. Additionally, we are examining sedentary behavior accumulated in bouts ≥ 30 min to capture the prolonged nature of sedentary behavior and associated health risks [25]. Finally, we also employ the ActiGraph Link as a sleep detection device to measure both duration and quality. Research in non-bariatric populations with obesity suggests that short sleep duration is a risk factor for weight gain and is improved via weight loss [26]; however, these relationships have received little attention in the bariatric population. The ActiGraph Link was chosen as a measurement tool for this project because it is tolerable to participants, unobtrusive, difficult to manipulate, and capable of wireless connectivity.

Smartphone surveys are used in conjunction with the ActiGraph Link to understand the context in which objectively measured physical activity and sedentary behaviors are occurring. When the ActiGraph Link detects that a MVPA bout lasting ≥10-min has concluded (as defined by ≥2 min below 3 METs), participants are prompted to self-report the type(s) of activities performed (walking, cycling, etc.) through PiLR. Likewise, participants are asked to self-report the type(s) of sedentary behaviors (watching TV, driving, etc.) performed. Four random prompts to complete a smartphone survey about sedentary behavior are administered per day between 10 a.m. – 12 p.m., 1 p.m.- 3 p.m., 4 p.m. – 6 p.m., and 7 p.m. – 9 p.m. If the participant is sedentary for 60 consecutive minutes (as defined by ≥2 min above 1.5 METs) without more than one minute of activity between sedentary minutes, then a random prompt is activated. However, if no prompt has been activated by the end of the window, then a survey is delivered at that time (e.g., 12 p.m., 3 p.m., 6 p.m., 9 p.m.). Smartphone surveys are also used to examine physical activity intentions and barriers through beginning- and end-of-day surveys.

### Eating behavior

The Bite Counter (Bite Technologies, Pendelton SC, USA) is a wrist-worn device that detects bites of food using an on-board tri-axial accelerometer to sense an upward arcing motion from table to mouth (i.e., “wrist roll”) thus tracking individual bites of food. The Bite Counter has been validated to detect bites taken during controlled eating in laboratory settings and free-living eating in adults [27, 28]. The number of bites determined by the Bite Counter has been well-correlated with estimated energy intake [29]. Using bite data, the device and accompanying software are validated to provide detailed information on patterns of eating (number, timing, duration, and rate of eating bouts) and approximate amount (in kilocalories) of food and drink ingested within and across daily eating bouts [28]. The Bite Counter has proven to be tolerable for participants to wear on a daily basis [30, 31].

Participants wear the Bite Counter on their dominant wrist for all waking hours during each 10-day assessment period (except time spent bathing, swimming, and charging the device). Participants are instructed to push a button on the device each time they begin eating to signal the device to begin collecting bite data. They push the same button when the eating episode has finished. For the current project, we sought to collect information in accordance with postoperative guidelines related to eating patterns [9]. As such, variables of interest are daily frequency, and duration of eating bouts, as well as bite count. The strengths of the Bite Counter are that it provides objective measurement on aspects of eating that are difficult to self-report (especially for individuals with overweight/obesity) [32], it is unobtrusive compared to other objective methods of assessing eating behavior, it was developed using sophisticated and systematic methods, and the display can be inactivated for assessment and activated for intervention purposes [33, 34].

For contextual factors related to eating that cannot be detected by sensor, questions regarding eating, appetitive experiences and attitudes are included in the 4 semi-random prompts to complete self-report surveys. The following factors are assessed: hedonic hunger (i.e., the drive to eat for pleasure rather than energy deficit), homeostatic hunger (i.e., the drive to eat due to prolonged food deprivation), satiety (i.e., the processes that inhibit further consumption in the postprandial period) and satiation (i.e., the processes that bring an eating episode to an end), dietary restraint (i.e., conscious efforts to restrict food intake to influence body weight and/or shape) [35], disinhibition (i.e., overeating in response to internal or external cues) [35], and grazing. End-of-day surveys include questions about binge eating, loss of control, consumption of high-fat/sugar foods, and bariatric complications (i.e., reflux, nausea, vomiting, diarrhea, cramping, bloating, dehydration, and fatigue).

#### Assessment of dietary intake

Dietary intake is measured during each assessment period via three non-consecutive, 24-h diet recalls representing two weekdays and one weekend day. During dietary recalls, participants are asked to recount all foods, beverages, and supplements consumed in the prior 24-h period. The first of the three recalls is collected at the in-person study visit, and the subsequent two recalls are collected over the phone. A trained interviewer collects the recalls using Nutrition Dietary Systems for Research (NDSR; Nutrition Coordinating Center, University of Minnesota, 2017). NDSR utilizes a multiple-pass interview approach, which provides the participant multiple opportunities to recall intake [36]. NDSR output files are used to characterize energy intake, percent energy from macronutrients, as well as micronutrient intake from the diet and supplements, separately. This output allows us to examine adherence to diet-specific surgical guidelines including: eating frequency, total energy intake, percent energy from macronutrients (protein, fat and carbohydrate), micronutrient intake (including supplements), and diet quality (as measured by the Healthy Eating Index, 2015 [37]). Regarding diet quality, the variable of interest for this particular population is the percent energy from empty calories (which includes calories from solid fats, added sugars and alcohol). As such, the percentage of empty calories is used to measure intake of “risky” foods. Dietary recalls are necessary as they are a valid and reliable method to study composition of dietary intake [38], which is not adequately measured by the Bite Counter alone.

### Other behavioral recommendations

Self-weighing facilitates weight loss and maintenance for non-bariatric patients but its importance in bariatric patients is less established [39]. Medication and supplement use is a focus of bariatric surgery guidelines [9]. Both are assessed in end-of-day surveys per our prior research [17].

### Psychosocial factors

These predictors are expected to relate to weight loss outcomes and may explain for whom and under what circumstances behaviors are related to weight loss outcomes due to prior evidence in the bariatric population and related health conditions. The following factors are being assessed during the 4 semi-random prompts: mood, stress, energy, and fatigue using items from the Profile of Mood States [40], Positive and Negative Affect Scale [41], and Daily Stress Inventory [42]; locus of control using items from the Multidimensional Health Locus of Control Scale [43]; and social support using items from the Multidimensional Scale of Perceived Social Support [44]. When possible, items were drawn from validated measures and adjusted for EMA administration if necessary.

### Environmental factors

While not yet emphasized in bariatric surgery guidelines, environmental factors may explain variability in weight loss outcomes via effects on behavior [45]. In this study, random survey prompts query the number and types of high quality palatable foods available, and other environmental characteristics of eating episodes studied in previous EMA studies including where eating occurs, where the food originated (e.g., prepared by self/other at home, restaurant, fast food), and the presence of others [17, 45–47]. Cues for physical activity (e.g., availability of exercise equipment and apparel) are also assessed during semi-random prompts.

### Paper and pencil questionnaires

Participants complete validated questionnaires to capture more general information on behaviors (e.g., physical activity, sedentary behavior, and eating) and clinical symptomatology (e.g., depression and anxiety symptoms). We also evaluate bariatric surgery expectations and perceptions (at baseline), as well as level of satisfaction with bariatric surgery outcomes (at each postoperative assessment) as these factors can impact adherence to dietary and health goals [48, 49].

### Chart review

Medical charts are reviewed after the study period is complete to collect data about attendance at clinical follow-up visits and patient support groups, types of postoperative care attended, (e.g., nutritional counseling), and adherence to clinical care regimen. Pre- and postoperative medical comorbidities are also extracted. Participants who drop-out provide consent for the research team to retrieve these data after discontinuing the study.

### Body weight and waist circumference

Participants’ body weight and waist circumference are measured at pre- and all postoperative assessments through 12 months. Weight is measured to the nearest 0.1 kg using a calibrated digital scale. Height is measured in millimeters using a wall-mounted Harnpenden stadiometer. From these measures, body mass index (kg/m^2^) is calculated. Postoperative weight loss is expressed in terms of kg and % weight loss. Waist circumference is measured at the midpoint between the highest point of the iliac crest and the lowest part of the costal margin in the mid-axillary line.

## Data monitoring and management

Prior to the start of the trial, a data safety monitoring plan was developed and approved by a pre-appointed Safety Officer. The Safety Officer, approved by the funding agency, is an expert in working with weight loss and bariatric surgery populations and has familiarity with the assessment tools employed in the current study. In the first 6 months of the project period (and annually thereafter), we have been submitting tables indicating our progress with recruitment, assessment retention, reasons for dropouts, and adverse events to the Safety Officer for review. After the review, the Safety Officer has provided written notice to the principle investigators and the funding agency as to whether the study is progressing appropriately and safely. Adverse events and serious adverse events are collected and reported from the beginning of study-related procedures to the end of the study follow-up period for each individual. At each visit, study staff specifically query participants for adverse events and participants are encouraged to report through telephone calls and emails as well. Should they occur, our policy is to report adverse events within 1–2 weeks to the IRB and serious adverse events are reported to the IRB and the funding agency within 24 h.

Our active data management plan involves cleaning the data, generating composite measures and performing data reduction activities with data collected via EMA, questionnaire, chart review and anthropomorphic measurement. Participant number identifies data collected and data is kept in locked files behind locked doors in the research centers. Additional safeguards are in place to protect participant data collected via sensor devices and electronic forms on smartphones. These data are stored temporarily on the smartphone but are regularly transmitted to encrypted secure storage on PiLR Health servers. Thus, if a study smartphone is lost or stolen, it is very unlikely that a participant’s confidential data would be compromised. Data transmitted via smartphones is also heavily encrypted by mobile phone carriers to prevent interception (e.g., from the smartphone to PiLR Health servers). No personally identifiable information is stored or transmitted via the smartphone. All participant smartphone data is coded using a unique identifying number. Any electronic data collected by study staff is stored in an encrypted form (with a randomly generated 26-character key).

## Statistical analysis

Aim 1 of this project seeks to develop and implement the EMA system and assess its feasibility and acceptability. The analytical plan for this aim is therefore descriptive in nature (e.g., examining compliance with wearing devices, completing survey prompts). Aim 2 seeks to examine which behavioral and psychosocial factors predict weight loss outcomes and the times at which each factor has the strongest effect on outcomes. Aim 2 will be evaluated using general longitudinal linear mixed effect models for weight loss and waist circumference as the dependent variables and baseline and time-varying behavioral, psychosocial, and environmental factors as predictors. Models will include the effect of control variables and covariates that are potential confounders between psychosocial and behavioral factors and weight loss outcomes, including: sex, race, ethnicity, and age. Aim 3 seeks to identify causal pathways by which psychosocial factors influence outcomes via effects on behavior; and moderators that explain for whom and under what conditions the influence is the strongest. To evaluate Aim 3, we will use the counterfactual approach to mediation modeling with exposure-mediator interaction (i.e., moderation) as described by Valeri and VanderWeele [50].

In all models, we will consider EMA and in-person measures of the same construct separately, and only together with the use of composites, to avoid multicollinearity. Patterns and potential causes of missingness will be evaluated. We plan to treat missing data as missing at random, to be addressed in analysis with multiple imputation and/or maximum likelihood parameter estimation [51].

Given the novelty of the current study, the sample size was selected in collaboration with the NIH as adequate to assess the feasibility of the study protocol and to also obtain reliable estimates of effect sizes to inform preliminary estimates and future studies. As our analysis plan is primarily descriptive, we note that a sample size of 100 is suitable for the detection of correlation coefficients that are small to moderate in magnitude (*r* > 0.28) as statistically significant using conventional type-I and type-II error probabilities (5 and 20%, respectively), and similarly group mean differences of 0.56 standard deviation units under similar assumptions and balance in representation across group. Such minimally detectable effect sizes are subtle enough to fall below conventional thresholds for clinical significance.

## Discussion

In line with the NIH focus on behavioral and psychosocial predictors of bariatric surgery outcomes, using a novel multi-sensor mHealth approach is expected to provide contextually rich data that can validate existing pre- and postoperative behavioral guidelines, inform new behavioral guidelines, and identify treatment targets for clinicians working with this population. The current study will be the first to capitalize on advancements in mHealth within the bariatric population by integrating multiple sensors and self-report methods to examine a variety of behavioral and psychosocial factors longitudinally over the pre- and postoperative period. As such, our methodology is expected to inform best practices in assessment for future studies of bariatric patients and produce data that will serve as a strong foundation for additional research within this population.

Our approach has many benefits over using solely traditional methodologies (i.e., chart review, self-report questionnaires) to study behavior in this population. EMA is, at present, one of the most valid and reliable tools for assessing individuals’ behaviors, thoughts, and emotions throughout the course of their daily lives in their natural environments. Our project extends the value of EMA by *combining and integrating* several EMA measurement methods (i.e., self-report surveys, actigraphy, and passively sensed eating) to inform a nuanced understanding of the contexts in which behaviors and psychosocial factors occur. In addition to enhanced accuracy and contextual validity, using an mHealth approach provides a temporal granularity that is paramount to understanding complex relationships between stable traits, changing states, and their influence on health behaviors [52]. For example, continuous data on behaviors of interest (e.g., physical activity, eating behavior) and repeated measurements of psychosocial factors (e.g., mood, environment, hunger) will allow us to understand the quasi-causal and reciprocal within-person associations between specific psychosocial factors and behaviors that impact surgical outcomes. In this vein, our multi-sensory methods are expected to generate a substantial amount of outcome data from which to answer a myriad of research questions related to bariatric surgery outcomes.

Another major strength of this study is that it was conceived and executed by a highly multidisciplinary research team including experts in behavioral science, bariatric surgery, clinical patient care, computer science, engineering, and business. Just as a multidisciplinary medical team is recommended to optimize care for bariatric surgery patients, our team approach has allowed us to develop a protocol and utilize assessment tools that are particularly well-suited to the needs of studying this complex population. Further, the multi-faceted expertise within our research team allowed us to use the challenges of this research to form novel interdisciplinary collaborations. For example, the tool used to objectively assess eating behavior in the current study (i.e., Bite Counter) requires a button press to start and stop monitoring of eating. This method likely introduced complications such as non-compliance with the button press and, in some individuals, reactivity. These complications created a need and opportunity to explore the viability of other methods for objectively assessing eating behavior. Thus, our team is now conducting research to use the ActiGraph Link (the device used for measuring physical activity, sedentary behavior, and sleep in our study) for continuous eating detection without button press using similar algorithms to the Bite Counter. This innovation is thought to enhance data quality and will allow for a single device to measure four different health behaviors (i.e., eating, activity, sedentariness, and sleep), which could reduce user burden and increase acceptability of protocol. Moreover, using the ActiGraph Link to passively sense eating behavior will allow for surveys to be triggered automatically when eating episodes are detected to better assess the physiological, psychological, and environmental factors related to eating (similar to the way in which the current protocol assesses physical activity). Finally, the combination of using both methods would allow for the validation of self-reported eating patterns collected via telephone dietary recalls.

There also have been general technical challenges to executing the current study that are inherent to any study utilizing mHealth methods. One major barrier was that our goal of continuously monitoring and integrating self-report surveys and sensor data is stretching the bounds of current technological capabilities. Much of a smartphone’s functionality is governed by a mobile operating system that dictates resource allocation to enable consistent functioning of multiple systems such as the touchscreen, Global Positioning System, Bluetooth, Wi-Fi, and camera. Typically, mobile operating systems work to conserve battery life of the device by limiting the degree to which software applications are allowed to run “in the background” (i.e., when they are not actively in use by the user or when the phone is in standby mode waiting to be used). This function makes it challenging to continuously synchronize sensor devices such as the ActiGraph Link with a smartphone and subsequently trigger self-report surveys in a timely manner as the corresponding applications are not permitted by the mobile operating system to run in the background. Mobile operating systems and software on sensor devices also tend to be updated by the manufacturer at unpredictable intervals and such updates often occur automatically outside of the control of the researchers. Such updates can disrupt a research study by causing mobile data collection systems to function unpredictably or cease functioning altogether. For this reason, it is important for researchers to have a plan (i.e., extra devices, back-up servers, contact with information technology teams) to maintain data collection systems and address problems caused by software updates.

Another way that we sought to mitigate these challenges was to increase our control of the technology by giving each participant the same type of smartphone for answering the self-report surveys and using research-grade sensors. However, as studies include longer monitoring periods and smartphones become more ubiquitous, it is becoming evident that the field should work towards deploying multi-sensor assessment methods that capitalize on the smartphones that many participants already own. This strategy would reduce the cost of conducting the study and lessen participant inconvenience, for example by eliminating the need for a participant to carry a personal smartphone and a research smartphone. The trade-off is that using personal smartphones would diminish control that the research team has over the tools used for data collection. Given that there are now a variety of mobile operating systems, using personal devices would also necessitate that data collection software to be interoperable (i.e., functional on a wide range of smartphone of smartphone operating systems). Many of the challenges experienced by our team in implementing mHealth tools reflect common barriers to conducting technology-based research in the field and call for deeper integration of information systems and behavioral science fields.

## Conclusion

As behavioral factors are increasingly recognized as contributors to bariatric surgery outcomes, the current study is critical for identifying factors and contexts that influence behavior among individuals who have undergone bariatric surgery. The project is designed to accumulate large quantities of data from both the pre- and postoperative period to evaluate a wide range of predictors of weight loss outcomes. Examples of potential research questions that will be tested using these data include: examining preoperative associations among behaviors and psychosocial factors; identifying preoperative predictors of outcome; demonstrating postoperative trajectories of weight and associated health behaviors; and systematically evaluating the that way in which various psychosocial factors can impact outcomes via changes in behavior. We plan to disseminate findings from the current study via peer-reviewed publication and conference presentations (no identifying participant information will be included in disseminated materials). Data and methods from the current study are also expected to provide a foundation for subsequent trials funded by NIH to examine behaviors that influence bariatric surgery outcomes. Overall, this program of research will contribute significantly to evidence-based clinical care for bariatric surgery patients.

## Additional file

Additional file 1: Sample Informed Consent Form from The Miriam Hospital IRB. (PDF 63 kb)

## Abbreviations

EMA: Ecological Momentary Assessment
IRB: Institutional Review Board
mHealth: Mobile health
MVPA: Moderate-to-vigorous physical activity
NDSR: Nutrition Data System for Research
RYGB: Roux-en-Y gastric bypass
SG: Sleeve gastrectomy
