# Supplementary material for: Multi-sensor ecological momentary assessment of behavioral and psychosocial predictors of weight loss following bariatric surgery: study protocol for a multicenter prospective longitudinal evaluation
Source: BMC Obes. 2018 Nov 5;5:27. doi: 10.1186/s40608-018-0204-6 (PMC6217766; doi:10.1186/s40608-018-0204-6)
Supplement: Supplementary file 1 — Sample Informed Consent Form from The Miriam Hospital IRB. (PDF 63 kb) [file 40608_2018_204_MOESM1_ESM.pdf]

Lifespan Affiliate Site where research will be conducted

☐ Rhode Island Hospital
☐ Bradley Hospital
☒ The Miriam Hospital
☐ Newport Hospital
☐ Gateway Healthcare

Agreement to Participate in a Research Study And Authorization for Use and Disclosure of Information

2112-15
Committee #

____
Name of Study Volunteer

Ecological Momentary Assessment of Behavioral and Psychosocial Predictors of Weight Loss Following Bariatric Surgery

You are being asked to take part in a research study. All research studies at Lifespan hospitals follow the rules of the state of Rhode Island, the United States government and Lifespan. Before you decide whether to be in the study, you and the researcher will engage in the "informed consent" process. During this process, the researcher will explain the purpose of the study, how it will be carried out, and what you will be expected to do if you participate. The researcher will also explain the possible risks and benefits of being in the study, and will provide other information. You should feel free to ask any questions you might have. The purpose of these discussions is for you to decide whether participating in the study is the best decision for you.

If you decide to be in the study, you will be asked to sign and date this form in front of the person who explained the study to you. This form summarizes the information you discussed. You will be given a copy of this form to keep.

1. Nature and Purpose of the Study: You are being asked to take part in a research project because you plan to have bariatric surgery. The purpose of the study is to determine whether weight-related behaviors (e.g., eating and physical activity patterns), psychological characteristics (e.g., mood and hunger), and environmental characteristics (e.g., supportiveness of family and friends) helps to explains who loses more versus less weight after bariatric surgery. We expect to enroll 100 participants in this study between the two sites at the Weight Control and Diabetes Research Center in Providence, Rhode Island and the Beth Israel Deaconess Medical Center in Boston, Massachusetts. The study is sponsored by the National Institutes of Health (NIH).

2. Explanation of Procedures: If you take part in this study, you will be asked to attend an in-person orientation session at the Weight Control and Diabetes Research Center of The Miriam Hospital and Brown University in downtown Providence, Rhode Island. During this orientation session, the research study will be explained to you in detail and you will have the opportunity to ask any questions that you may have. If you decide to enroll, you will complete this consent form with a member of the research team. Your height and weight will then be measured. You will be asked to complete an initial 24 hour diet recall and questionnaires related to your eating and physical activity habits and experiences. Some questionnaires also contain questions about your mood, your ability to manage your health, supportiveness of your family and friends, and your expectations for bariatric surgery. You will also complete two additional 24-hour diet recalls over

the phone during the assessment period.

You will also be given three devices used in "Ecological Momentary Assessment" (EMA) to measure your weight-related behaviors and experiences for 10 days:

ActiGraph Link: This commercially available device measures your physical activity and the amount of time that you spend sitting or lying down, and sleeping. It has the size and appearance of a large wristwatch. You will be asked to wear this device on your wrist for 10 days except for when you are bathing or swimming. This device may be used to send the data that it collects to the smartphone provided to you for research purposes via a wireless connection. The smartphone may then send the data to the researchers whenever it has a connection to the cellular network. Therefore, the researchers may have access to near immediate information on your physical activity, sitting or lying down, and sleep.

Bite Counter: This commercially available device looks like a wristwatch and is worn on the wrist of the arm that you use for eating. It keeps track of when and how many bites that you take when you eat food. You will be asked to wear this device during your waking hours. You will be asked to plug it in to charge when you are sleeping. This device may be used to send the data that it collects to the smartphone provided to you for research purposes via a wireless connection. The smartphone may then send the data to the researchers whenever it has a connection to the cellular network. Therefore, the researchers may have access to near immediate information on your eating behavior.

Smartphone: This device will be used to ask you questions about your current weight-related behaviors and experiences as they are happening. You may be asked to answer questions about the following:

- Your eating behavior including the types of foods that you eat, the source of the food (e.g., prepared at home, restaurant) the types of foods that are around you when you are eating, where you eat, who is with you when you eat;
- Your appetite, hunger, feelings of control over eating, feelings of fullness after eating;
- Problematic eating behavior such as grazing and binge eating;
- Your physical activity behavior;
- Your intentions to be physically active and barriers to physical activity;
- Activities performed while sitting or lying down (e.g., working, reading, watching TV).
- Your mood, level of stress, feelings of energy and fatigue;
- The degree to which you feel in control of your health;
- The degree to which the people around you support you in making healthy choices;
- Whether or not you weigh yourself.
- Behavioral and gastrointestinal complications related to bariatric surgery including reflux, nausea, vomiting, diarrhea, cramping, bloating, dumping syndrome, and dehydration.

The smartphone will beep and vibrate when it is time for you to answer questions. The questions will take up to 5 minutes to answer each time. You may be asked to answer questions several times per day. If you are not able to answer questions right away, you can answer them again later. The smartphone will transmit your answers to the research team, but the research team will not review them immediately. <u>If you experience any problems with your health or behaviors, you should contact your medical providers immediately; the research team will not contact you about your</u>

answers to the questions. If you do not wear the Actigraph Link or Bite Counter, or if you miss more than one 1 of every 10 sets of questions, you may be contacted by the research team by phone and/or email to ask if there is a problem and to encourage you to use the devices as instructed.

Once you have completed 10 full days of EMA, you will be asked to return the three devices to the Weight Control and Diabetes Research Center. You will be asked to return to the Weight Control and Diabetes Research Center again 3 months, 6 months, and 12 months after your bariatric surgery to repeat height and weight measurement the three 24-hour diet recalls, questionnaires, and EMA. If you follow the study procedures, you will receive $75 for each assessment before surgery, and at 3 months, 6 months, and 12 months after your bariatric surgery. You will also receive 50 cents for each survey you submit on the smartphone totaling up to an additional $25 at each assessment.

Information will be collected from you and transmitted via the cellular network as part of this research study. Data from the ActiGraph Link and Bite Counter devices, as well as your answers to questions presented on the smartphone, may be transmitted via the cellular network to research staff. Lifespan takes your confidentiality seriously and will take steps to protect this information to the degree permitted by the technology being used. In this study, the following steps will be taken: encrypting the data during transmission, limiting sensitive health care information collected and transmitted via the cellular network, storing all data gathered on secure servers, providing you with a secure device, and remote data deletion in the event of a lost or stolen device.

However, Lifespan can make no guarantees about the secure transmission of information collected from you. For example, your answers to questions that display on the smartphone screen may be seen by someone close by. Also, if the smartphone is lost or stolen, someone may be able to gain access to your most recent answers to questions on the smartphone. To try to lessen these risks, we collect your information from the smartphone whenever there is a cellular connection so that very little of your information is stored on the smartphone at any given time. We also suggest that you only answer questions on the smartphone where no one will be able to view the screen.

As part of this study, the research team will also ask your bariatric surgery provider for the dates that you attended visits for follow-up care and/or support groups after bariatric surgery. This information will be taken from your medical record.

There is no cost for participating in this study. This study does not pay for any of your medical care.

Contact Information: You may contact Dr. Graham Thomas at (401) 793-8154 with any questions or concerns about this study.

3. Discomforts and Risks: The risks involved in this study are minimal. None of the questions asked in this study are considered sensitive, but you may find that you do not like to answer the questions.

4. Benefits: There are no direct benefits to you for participating in the study other than the opportunity to contribute to science in a way that may improve care for future bariatric surgery patients.

5. Alternative Therapies: This study does not involve treatment of any kind.

6. Refusal/Withdrawal: It is up to you whether you want to be in the study. You are not required to enroll or participate. If you decide to participate, you can always change your mind and quit at any time. If you decide not to be in the study, or if you quit later, you will still be able to get the health care services you normally get. If you join, but later on the researcher or your doctor feels being in the study is no longer good for you, they may choose to take you out of the study before it is over. If new information becomes available that might change your mind about whether you want to stay in the study the researcher will share this information with you as soon as possible.

Follow-up after Withdrawal of Consent

If you leave the study, it would still be useful for us to know how you do until a year after your bariatric surgery. We would appreciate if you would permit us to get follow-up information about your body weight from your doctor or your medical record.

____ If I withdraw from the study, you have my permission to collect information about my body weight from my doctor or medical record.

_____ I do not give my permission for you to continue to collect information about me if I stop participating in the study.

____________________________________ ____________
Signature of study volunteer Date

You have the right to change your mind at any time regarding follow-up after withdrawal. If you decide to quit the study please tell the head researcher Dr. Graham Thomas at (401) 793-8154.

7. Medical Treatment/Payment in Case of Injury: A research injury is any physical or mental injury or illness caused by being in the study. If you are injured by a medical treatment or procedure you would have received even if you were not in the study that is not a research injury. To help avoid research injury and added medical expenses, it is very important to follow all study directions carefully. If you do experience a research injury, Lifespan or the study doctor can arrange medical treatment for you. Such treatment will be paid for as described below.

If you have insurance and have a research injury that is not covered by the study, it is possible that some or all of the cost of treating you could be billed to your insurer. If your health insurance will not cover such costs, it is possible you would have to pay out of pocket. In some cases, Lifespan might be able to help you pay if you qualify for free care under Lifespan policy. However, Lifespan has no policy to cover payment for such things as lost wages, expenses other than medical care, or pain and suffering.

8. Rights and Complaints: Signing this form does not take away any of your lawful rights. If you have any complaints about this study, or would like more facts about the rules for research studies, or the rights of people who take part in research studies you may contact Janice Muratori in the Lifespan Office of Research Administration, at (401) 444-6246

9. Confidentiality and Research Authorization for Use and Disclosure of Your Health Care Information: Your research records will be treated as private health care records and will be protected according to Lifespan privacy practices and policies that are based on state and federal law. In particular, federal law requires us to get your permission to use or disclose (release your information to someone outside of Lifespan) your health information for research purposes. If you sign this form you agree to be in this research study and you permit the use and disclosure of your health information for the purpose of conducting the research, providing treatment, collecting payment and running the business of the hospital. This permission has no expiration date. You may withdraw from the study at any time. However, if you do not want the researchers to use or disclose any further information in this study you must cancel permission in writing and may do so at any time. If you cancel your permission, you will stop taking part in the study and no new information will be collected about you. However, if you cancel your permission, it will not apply to actions already taken or information already collected about you by the hospital or the researchers before you canceled your permission.

Generally, the entire research record and any medical records held by the hospital may be used and released for research purposes. The following people or businesses/companies/ might use, release, or receive such information:

- The researcher and their support staff;
- The study sponsor: The National Institutes of Health (NIH)
- Doctors, nurses, laboratories and others who provide services to you or the sponsor in connection with this study;
- The company or section of the U.S. government that is paying for the study and others they hire to oversee, administer, or conduct the research;
- The United States Food and Drug Administration, the Department of Health and Human Services, the Office of Inspector General, and the Office of Civil Rights; European Medicines Agency
- People who volunteer to be patient advocates or research volunteer protectors;
- Members of the hospital's administrative staff responsible for reviewing, approving and administering clinical trials and other healthcare or research activities.
- Accrediting Organizations

There are times when the law might require or permit Lifespan to release your health information without your permission. For example, Rhode Island law requires researchers and health care workers to report abuse or neglect of children to the Department of Children, Youth and Families (DCYF) and to report abuse or neglect of people age 60 and older to the Department of Elderly Affairs.

All researchers and health care providers are required to protect the privacy of your health care information. Other people and businesses/organizations that are not health care providers are not required by law to do that so it is possible they might re-release your information.

You have the right to refuse to sign this form and not participate in the research. Your refusal would have no affect on your treatment, charges billed to you, or benefits at any Lifespan health care site. If you do not sign, you will not be able to enroll in the research study and will not receive treatment as a study participant.

If you decide to quit the study after signing this form (as described in Section 6) no new information will be collected about you unless you gave us permission to do so. However, the hospital or the researchers may continue to use information that was collected before you quit the study to complete analysis and reports of this research.

For more detail about your privacy rights see the Lifespan Joint Privacy Notice which has or will be given to you.

____

Study Volunteer Initials

SIGNATURE

I have read this informed consent and authorization form. ALL OF MY QUESTIONS HAVE BEEN ANSWERED, AND I WANT TO TAKE PART IN THIS RESEARCH STUDY.

By signing below, I give my permission to participate in this research study and for the described uses and releases of information. *I also confirm that I have been now or previously given a copy of the Lifespan Privacy Notice*

This informed consent document expires on 07/23/2018.
DO NOT sign this document after this expiration date

The Researcher is required to provide a copy of this consent to you.

____ ________ ____________

Signature of study volunteer/authorized representative* Date and Time when signed

I WAS PRESENT DURING THE CONSENT PROCESS AND SIGNING OF THIS AGREEMENT BY THE STUDY VOLUNTEER OR AUTHORIZED REPRESENTATIVE

____ ______________

Signature of witness (required if consent is presented orally or at the request of the IRB) Date

____ ______________

Signature of Translator Date

____ ________ __________

Signature of researcher or designate Date and Time when signed

* If signed by agent other than study volunteer, please explain below.

____

____
